# Comparative effectiveness of orthokeratology versus highly aspherical lenslets in slowing myopia progression: a retrospective study

**DOI:** 10.1186/s40662-026-00505-0

**Published:** 2026-08-15

**Authors:** Minfeng Chen, Xianling Yang, Huihui Lei, Jiayi Song, Yue Tang, Xiaochi Chen, Zhanmei Du, Menghua Han, Xinjie Mao

**Affiliations:** National Clinical Research Center for Ocular Diseases, Eye Hospital, Wenzhou Medical University, Wenzhou, 325027 China

**Keywords:** Axial length, Myopia control, Orthokeratology, Highly aspherical lenslets

## Abstract

**Background:**

To compare the axial length (AL) elongation between orthokeratology (OK) and highly aspherical lenslets (HAL) in children with myopia.

**Methods:**

This retrospective study included children with spherical equivalent refraction (SER) ranging from − 0.50 to − 9.00 D who were treated with OK lenses or HAL spectacles. Propensity score matching (PSM) was performed to balance baseline characteristics, including age, SER, sex, and baseline AL. The primary outcome was the 12-month AL elongation. Subgroup analyses were conducted according to age (8–9, 10–11, and 12–14 years) and refractive error categories (mild: − 0.50 to − 3.00 D; moderate: − 3.00 to − 5.00 D; high: − 5.00 to − 9.00 D).

**Results:**

After PSM, 3,122 matched pairs (n = 6,244) with balanced baseline characteristics were included. Overall, OK group had greater AL elongation over 12 months than the HAL group (0.20 ± 0.18 mm vs. 0.15 ± 0.17 mm; *P* < 0.001). In age-stratified analyses, the OK group demonstrated greater AL elongation than the HAL group among younger children, including those aged 8–9 years (0.26 ± 0.18 mm vs. 0.19 ± 0.18 mm;* P* < 0.001) and 10–11 years (0.17 ± 0.17 mm vs. 0.12 ± 0.17 mm; *P* < 0.001). By contrast, no significant between-group difference was observed in children aged 12–14 years (*P* = 0.305). SER-stratified analyses revealed a refractive-error-dependent pattern. In children with mild myopia (− 0.50 to − 3.00 D), the OK group demonstrated greater AL elongation than the HAL group (*P* < 0.001). By contrast, among children with high myopia (− 5.00 to − 9.00 D), the OK group had lower AL elongation than the HAL group (*P* < 0.001), while no significant difference was observed for moderate myopia (*P* = 0.950). Subgroup analyses were performed using a propensity score-matched cohort.

**Conclusions:**

HAL were associated with less AL elongation in younger children and those with mild myopia, whereas OK lenses were associated with less AL elongation in children with high myopia. No significant differences between treatment modalities were observed in children with moderate myopia or in older children.

## Background

The global myopia epidemic has emerged as a critical public health priority owing to its increasing prevalence and associated complications, which pose substantial socioeconomic burdens. Current epidemiological projections indicate that without effective intervention strategies, 49.8% of the world’s population will be affected by myopia by 2050, including 9.8% with high myopia [1]. Of particular concern is the trend toward an earlier age of myopia onset, with an increasing proportion of early-onset cases reported in recent decades [2]. This demographic shift has precipitated a parallel rise in the prevalence of high myopia, elevating population-attributable risks of vision-threatening complications, including retinal detachment, myopic maculopathy, and glaucoma [1–3].

Current myopia control strategies have varying efficacy profiles [4–7]. Orthokeratology (OK) lenses reduce axial length (AL) elongation by 36%–56% compared with single-vision spectacles in children with mild-to-moderate myopia [4, 8–15]. Highly aspherical lenslets (HAL) have achieved comparable efficacy, with Bao et al. reporting 55% and 51% reductions in spherical equivalent progression and AL elongation, respectively, over 24 months [15, 16]. Although both modalities demonstrate well-established myopia control effects in mild-to-moderate myopia, the comparative efficacy of OK and HAL in controlling high myopia remains insufficiently characterized in the existing clinical evidence. Conventional OK lenses are primarily used for mild-to-moderate myopia. Their application in high myopia presents two clinical challenges: uncorrected residual refractive error and suboptimal daytime visual acuity after lens removal. A novel dual-correction approach combining overnight OK with daytime spectacles has demonstrated the potential to address these limitations by maintaining both optimal visual correction and the established myopia control benefits of OK [17].

Emerging evidence from previous studies demonstrates a positive correlation between baseline myopia severity and treatment efficacy for OK, with superior AL elongation control observed in children with moderate and high myopia compared to mild cases [4–7]. However, the dose–response relationship for HAL remains unclear. Specifically, the effects of HAL on myopia control have only been reported in children with mild-to-moderate myopia. Whether HAL offers good myopia control in highly myopic children remains unclear [15, 16]. Furthermore, most studies to date have been conducted under controlled conditions in randomized controlled trials. However, this may not fully reflect real-world clinical settings. Importantly, research comparing the effectiveness of different optical interventions for myopia control in real-world clinical settings is limited, and the best approach for myopia control in children with varying degrees of refractive error remains elusive.

This retrospective study aimed to compare AL elongation between OK and HAL in children with mild, moderate, and high myopia in a real-world setting. The study also analyzed the annual AL elongation across different myopia levels to explore how the relative differences between treatments varied with the baseline refractive error.

## Methods

### Research participants

Data of 26,308 children with myopia who underwent OK or HAL fitting at the Eye Hospital of Wenzhou Medical University between January 2020 and March 2024 were retrospectively analyzed. The inclusion criteria were as follows: (1) age of 8–14 years; (2) spherical equivalent refraction (SER), − 0.50 to − 9.00 diopters (D) with cylindrical power ≤ 2.00 D; (3) best-corrected visual acuity ≥ 0.1 logMAR in both eyes; (4) no ocular or systemic comorbidities; (5) no history of myopia control treatment (including low-concentration atropine) within the preceding year; and (6) absence of strabismus, amblyopia, or binocular vision dysfunction. Data on age, sex, visit time, prescription, and eyewear purchase history were extracted from the clinical electronic medical record system. After applying stringent inclusion criteria and ensuring complete baseline and 12-month follow-up data (including AL, corneal curvature, and subjective refraction), 7,794 participants (OK: n = 3,498; HAL: n = 4,296) were included in the final analysis. To mitigate the confounding bias arising from baseline differences between the two groups, propensity score matching (PSM) was employed to achieve a baseline balance. A binary logistic regression model was constructed with group assignment (HAL and OK) as the dependent variable and incorporating age, spherical equivalent, sex, and baseline AL as covariates to estimate the propensity score for each participant. Subsequently, 1:1 nearest-neighbor matching without replacement was performed, with the caliper width set to 0.02. After PSM, 6,244 participants (3,122 in the OK group, 3,122 in the HAL group) were included in the analysis.

**Fig. 1:**
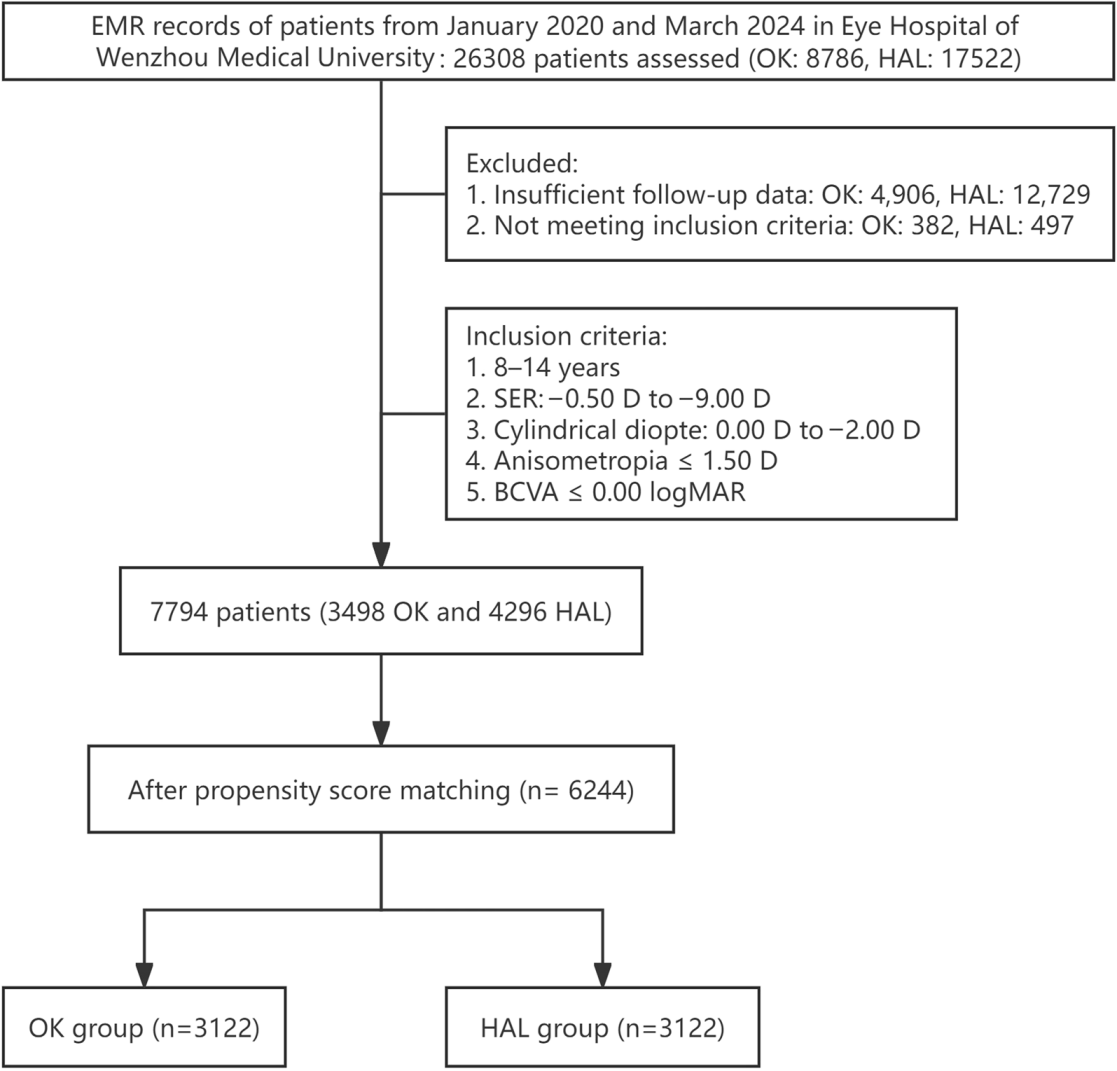
Flowchart of propensity score matching (PSM). This figure illustrates the inclusion and exclusion processes of the participants from the electronic medical record (EMR) system. A total of 26,308 children with myopia were screened, and 7,794 met the eligibility criteria. After PSM based on age, spherical equivalent refraction (SER), sex, and baseline axial length, 3,122 matched pairs (n = 6,244) were included in the final analysis. HAL, highly aspherical lenslet; OK, orthokeratology

AL elongation after 1 year of lens wear was compared between the two groups across age, refractive error, and sex subgroups (Fig. 1, showing study inclusion flowchart). The study protocol was approved by the Institutional Review Board of the Eye Hospital of Wenzhou Medical University (2024-135-K-118-01) in accordance with the Declaration of Helsinki, and the requirement for informed consent was waived for the analysis of de-identified electronic medical records.

### Instrumentation

OK lenses (Euclid, Paragon CRT, DreamLite, #ORTHOK, LUCID, and DreamVision) were fitted by experienced ophthalmologists according to the manufacturer’s protocols. Before fitting, participants underwent a comprehensive baseline examination including medical history, visual acuity, slit-lamp and fundus examinations, autorefraction and subjective refraction, keratometry, corneal topography, intraocular pressure measurement, and AL measurement. The participants were instructed to wear lenses nightly for at least 8 h. Follow-up visits were scheduled at 1 day, 1 week, 1 month, and every 2–3 months thereafter, during which visual acuity, slit-lamp examination, refraction, and corneal topography were assessed. For children with high myopia who underwent OK, supplementary single-vision spectacles were prescribed during the daytime to correct residual refractive errors after overnight OK treatment [17, 18].

Participants in the HAL (Stellest^®^, EssilorLuxottica, Charenton-le-Pont, France) group were advised to wear the spectacles daily for at least 12 h. Follow-ups were scheduled at 3–6-month intervals, with assessments of visual acuity, slit-lamp examination, and refraction. AL was measured every 3 or 6 months. The AL measurements ultimately included for analysis in the HAL and OK groups were those obtained at 12 ± 1 months, in order to assess the 1-year AL elongation. The mean follow-up duration was 11.80 months in the HAL group and 11.47 months in the OK group. Since the refractive power in the OK group fluctuated daily after lens removal, the subjective refraction results were not entirely accurate. Moreover, some participants in the OK group did not undergo subjective refraction examination. Consequently, AL changes were ultimately used to evaluate myopia progression and changes in refractive power were not analyzed. Differences in AL changes between the two groups were compared according to various subgroups, including age subgroups (8–9, 10–11, 12–14 years), SER subgroups (mild myopia: − 0.50 D < SER ≤ − 3.00 D; moderate myopia: − 3.00 D < SER ≤ − 5.00 D; high myopia: − 5.00 D < SER ≤ − 9.00 D), sex subgroups, and astigmatism subgroups (mild astigmatism: − 0.00 D to − 0.50 D; moderate astigmatism: − 0.50 D to − 2.00 D, exclusive of − 0.50 D). All analyses were based on data obtained from the right eye.

For all participants, AL was measured using the IOLMaster 500 (Carl Zeiss), with a mean of five measurements used for the analysis. Individual measurements required a signal-to-noise (SNR) ratio > 2.0, and a composite SNR > 32.

### Statistical methods

Statistical analyses were performed using the SPSS software (version 24.0; IBM Corp., Armonk, NY, USA). Continuous variables were assessed for normality using the Shapiro–Wilk test and are presented as means ± standard deviations. PSM was performed to mitigate confounding bias arising from baseline differences between the two groups. A binary logistic regression model was constructed with group assignment (HAL vs. OK) as the dependent variable and incorporating age, SER, sex, and baseline AL as covariates to estimate the propensity scores. Subsequently, 1:1 nearest-neighbor matching without replacement was performed using a caliper width of 0.02. Participants without suitable matches within a specified caliper were excluded. Standardized mean differences (SMDs) were used to assess covariate balance before and after matching, with |SMD|< 0.1 indicating adequate balance. Between-group comparisons were performed using independent-sample t-tests for continuous variables. Paired t-tests were used to assess within-group changes in the AL from baseline to 12 months. Effect sizes are reported as mean differences. Pearson’s correlation analysis was used to evaluate the association between baseline variables and AL elongation. Multivariate linear regression analysis was performed to identify independent factors associated with AL elongation, including age, sex, SER, and baseline AL as covariates. PSM was used to reduce potential confounding due to nonrandom treatment allocation, and subsequent multivariable regression analysis was performed to further adjust for residual confounding and identify independent factors associated with AL elongation. Only participants with complete baseline and follow-up data were included in the analysis; therefore, missing data were not imputed. Statistical significance was defined as a two-sided *P* < 0.05.

## Results

### General information

From an initial pool of 26,308 children with myopia, 7,794 were enrolled. Propensity scores were estimated using a binary logistic regression model that included age, spherical equivalent, sex, and baseline AL as covariates. Subsequently, 1:1 nearest-neighbor matching without replacement was performed using a fixed caliper width of 0.02. This process successfully yielded 3,122 matched pairs, totaling 6,244 participants; the remaining 1,550 participants were excluded because of the absence of suitable matches within the specified caliper range.

**Fig. 2:**
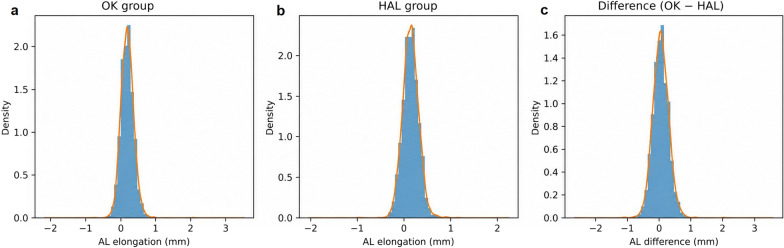
Distribution of axial length (AL) elongation. **a** Distribution of AL elongation in the OK group. **b** Distribution of AL elongation in the HAL group. **c** Distribution of the difference in AL elongation between groups (OK–HAL). Histograms with kernel density estimates are shown. The distributions were approximately normal with comparable variability across groups. HAL, highly aspherical lenslets; OK, orthokeratology

Before matching, substantial baseline imbalances were observed between the two groups. The SMDs for age, SER, baseline AL, and sex were − 0.263, 0.273, − 0.166, and − 0.182, respectively. Specifically, compared to the OK group, the HAL group exhibited a younger mean age, smaller absolute spherical equivalent, slightly shorter mean baseline AL, and a lower proportion of female participants. After PSM, each group comprised 3,122 participants. The SMDs for age, SER, baseline AL, and sex were substantially reduced to 0.012, − 0.001, 0.006, and 0.037, respectively, all below the threshold of 0.1, indicating excellent baseline balance after matching (Table 1). The distributions of AL elongation in the OK and HAL groups as well as the distribution of the between-group differences were approximately normal, with comparable variability, supporting the use of parametric statistical analyses (Fig. 2).

**Table 1:** Baseline demographics and characteristics of between two groups

Characteristic	Before propensity score matching					After propensity score matching				
	OK	HAL	T	*P*	SMD	OK	HAL	T	*P*	SMD
Sample size, n	3498	4296				3122	3122			
Male/female	1594/1904	2332/1964			− 0.182	1484/1638	1426/1696			0.037
Age (years)	10.15 ± 1.73	9.71 ± 1.62	11.512	< 0.001	− 0.263	10.06 ± 1.59	10.04 ± 1.71	0.471	0.638	0.012
SER (D)	− 3.06 ± 1.35	− 2.66 ± 1.57	− 12.160	< 0.001	0.273	− 2.99 ± 1.59	− 2.99 ± 1.34	− 0.022	0.982	− 0.001
AL (mm)	24.86 ± 0.86	24.71 ± 0.95	7.095	< 0.001	− 0.166	24.83 ± 0.97	24.83 ± 0.86	− 0.237	0.812	0.006

*AL* = axial length; *D* = diopter; *HAL* = highly aspherical lenslets; *n* = number of participants; *OK* = orthokeratology; *SER* = spherical equivalent refraction; *SMD* = standardized mean difference

Data are presented as number (n) or as mean ± standard deviation

The mean AL elongation was 0.1467 mm in the HAL group and 0.1919 mm in the OK group before matching. After matching, the corresponding values were 0.1468 and 0.1964 mm, respectively. Both analyses demonstrated a smaller AL elongation in the HAL group than in the OK group. Further regression analyses revealed that after covariate adjustment in the unmatched sample, HAL wear was associated with 0.0549 mm less AL elongation than OK wear (*P* < 0.001). In the matched sample, after additional adjustment, the estimated between-group difference remained at 0.0490 mm (*P* < 0.001). The consistent direction and comparable magnitude of the effect estimates derived from both analytical approaches suggest that the PSM procedure did not materially alter the primary study conclusions, supporting the robustness of the matching strategy.

### Comparison of AL elongation between groups across subgroups

Overall, children wearing OK lenses exhibited significantly greater AL elongation after 1 year than those wearing HAL spectacles (0.20 ± 0.18 mm vs. 0.15 ± 0.17 mm, *P* < 0.001).

Subgroup analyses demonstrated that the relative differences between the treatment modalities varied according to age and refractive error (Table 2). HAL was associated with significantly less AL elongation than OK in younger children (8–11 years) and in those with mild myopia. By contrast, OK was associated with lower AL elongation in children with high myopia. No significant between-group differences were observed among children aged 12–14 years or those with moderate myopia. Significant differences between the treatment modalities were also observed across the sex and astigmatism subgroups (Table 2).

**Table 2:** Comparison of AL elongation between the OK and HAL groups across subgroups

Characteristic	OK		HAL		T	*P*
	n (%)	AE (mm)	n (%)	AE (mm)		
Total	3122	0.20 ± 0.18	3122	0.15 ± 0.17	11.019	< 0.001
Age group						
8–9 years	1382 (44.3%)	0.26 ± 0.18	1310 (42.0%)	0.19 ± 0.18	10.802	< 0.001
10–11 years	1067 (34.2%)	0.17 ± 0.17	1138 (36.5%)	0.12 ± 0.17	6.674	< 0.001
12–14 years	673 (21.6%)	0.10 ± 0.16	674 (21.6%)	0.11 ± 0.14	− 1.027	0.305
F		217.649		66.317		
*P*		< 0.001		< 0.001		
Refractive status						
Mild myopia	1772 (56.8%)	0.23 ± 0.18	1836 (58.8%)	0.13 ± 0.18	16.229	< 0.001
Moderate myopia	1110 (35.6%)	0.17 ± 0.18	938 (30.0%)	0.17 ± 0.16	0.063	0.950
High myopia	228 (7.3%)	0.09 ± 0.17	345 (11.1%)	0.19 ± 0.15	− 6.835	< 0.001
F		72.386		30.813		
*P*		< 0.001		< 0.001		
Sex						
Female	1638 (52.5%)	0.20 ± 0.19	1696 (54.3%)	0.15 ± 0.18	7.917	< 0.001
Male	1484 (47.5%)	0.19 ± 0.18	1426 (45.7%)	0.14 ± 0.16	7.772	< 0.001
t		2.021		2.073		
*P*		0.043		0.038		
Astigmatism status						
Mild astigmatism	2319 (74.3%)	0.20 ± 0.18	2183 (69.9%)	0.14 ± 0.17	11.746	< 0.001
Moderate astigmatism	803 (25.7%)	0.19 ± 0.20	939 (30.1%)	0.17 ± 0.16	2.207	0.027
t		1.404		− 4.882		
*P*		0.161		< 0.001		

*AE* = axial length elongation; *AL* = axial length; *D* = diopter; *HAL* = highly aspherical lenslets; *n* = number of participants; *SER* = spherical equivalent refraction; *OK* = orthokeratology

Mild myopia: − 0.50 D < SER ≤ − 3.00 D; moderate myopia: − 3.00 D < SER ≤ − 5.00 D; high myopia: − 5.00 D < SER ≤ − 9.00 D; mild astigmatism: 0.00 D to − 0.50 D; moderate astigmatism: − 0.50 D to − 2.00 D, exclusive of − 0.50 D

### Comparative analysis of AL elongation in children with different degrees of myopia using various correction methods.

**Fig. 3:**
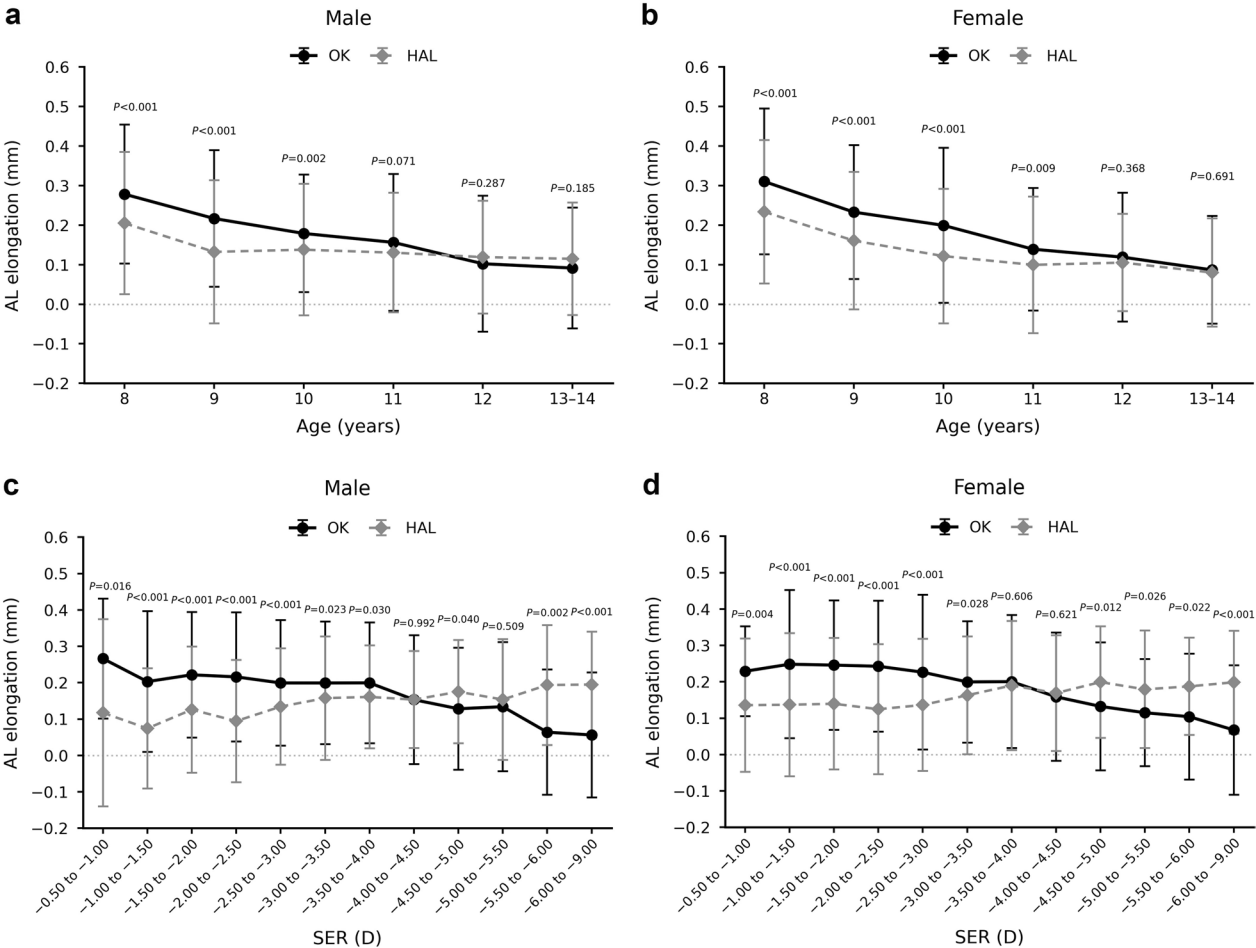
Axial length (AL) elongation in the OK and HAL groups stratified by age, sex, and SER. This figure presents subgroup comparisons of AL elongation over 12 months between children treated with OK and HAL (age-specific comparisons in males and females, showing AL elongation across age subgroups (8–9, 10–11, and 12–14 years; SER-dependent comparisons in males and females, illustrating AL elongation across refractive error categories (mild: − 0.50 to − 3.00 D; moderate: − 3.00 to − 5.00 D; high: − 5.00 to − 9.00 D). HAL, highly aspherical lenslets; OK, orthokeratology; SER, spherical equivalent refraction

Subgroup analyses revealed that the relative efficacies of HAL and OK lenses varied with age and refractive error. Among children aged 8–11 years, HAL was associated with a significantly slower rate of AL elongation than OK was. Conversely, no significant intergroup differences were observed in the older cohort (12–14 years). Analysis by refractive error demonstrated a nuanced, severity-dependent pattern: HAL was associated with less AL elongation for mild myopia (− 1.00 D to − 3.00 D), whereas OK became progressively more effective for higher myopia, showing a significant advantage beyond − 5.00 D. These differential treatment effects were consistent across sexes (Fig. 3). Table 3 presents the AL changes and Fig. 3 illustrates the trends following HAL and OK treatments across different sex, age, and refractive error subgroups.

**Table 3:** Comparative analysis of AL elongation (mm) in myopic children with different degrees of myopia and different age in the two groups

SER (D)		Age (years)					
		8	9	10	11	12	13–14
Male							
− 0.50 to − 1.875	OK	0.32 ± 0.02	0.23 ± 0.02	0.18 ± 0.02	0.19 ± 0.02	0.12 ± 0.03	0.13 ± 0.03
	HAL	0.15 ± 0.02	0.10 ± 0.02	0.09 ± 0.02	0.09 ± 0.03	0.08 ± 0.02	0.07 ± 0.04
− 2.00 to − 2.875	OK	0.27 ± 0.02	0.26 ± 0.02	0.21 ± 0.02	0.15 ± 0.02	0.14 ± 0.02	0.09 ± 0.02
	HAL	0.21 ± 0.02	0.10 ± 0.02	0.09 ± 0.02	0.10 ± 0.02	0.08 ± 0.02	0.08 ± 0.03
− 3.00 to − 3.875	OK	0.27 ± 0.02	0.21 ± 0.02	0.20 ± 0.02	0.16 ± 0.02	0.10 ± 0.03	0.15 ± 0.03
	HAL	0.26 ± 0.03	0.14 ± 0.02	0.18 ± 0.02	0.14 ± 0.02	0.13 ± 0.02	0.11 ± 0.02
− 4.00 to − 4.875	OK	0.23 ± 0.03	0.17 ± 0.02	0.13 ± 0.03	0.12 ± 0.03	0.06 ± 0.03	0.10 ± 0.02
	HAL	0.28 ± 0.04	0.20 ± 0.03	0.17 ± 0.02	0.14 ± 0.02	0.15 ± 0.02	0.12 ± 0.02
− 5.00 to − 5.875	OK	0.26 ± 0.03	0.07 ± 0.04	0.10 ± 0.06	0.13 ± 0.03	0.06 ± 0.04	− 0.03 ± 0.03
	HAL	0.14 ± 0.07	0.22 ± 0.05	0.19 ± 0.05	0.17 ± 0.03	0.15 ± 0.03	0.10 ± 0.04
− 6.00 to − 9.00	OK	0.16 ± 0.09	0.17 ± 0.06	0.08 ± 0.07	0.10 ± 0.07	0.00 ± 0.05	0.03 ± 0.07
	HAL	0.25 ± 0.08	0.23 ± 0.06	0.22 ± 0.04	0.20 ± 0.03	0.17 ± 0.03	0.40 ± 0.16
Female							
− 0.50 to − 1.875	OK	0.32 ± 0.02	0.25 ± 0.02	0.20 ± 0.02	0.18 ± 0.02	0.18 ± 0.03	0.17 ± 0.03
	HAL	0.20 ± 0.02	0.15 ± 0.02	0.08 ± 0.02	0.08 ± 0.02	0.08 ± 0.03	0.02 ± 0.03
− 2.00 to − 2.875	OK	0.34 ± 0.02	0.26 ± 0.02	0.22 ± 0.02	0.20 ± 0.02	0.12 ± 0.02	0.12 ± 0.03
	HAL	0.22 ± 0.02	0.15 ± 0.02	0.10 ± 0.02	0.04 ± 0.02	0.09 ± 0.03	0.03 ± 0.03
− 3.00 to − 3.875	OK	0.30 ± 0.02	0.23 ± 0.02	0.20 ± 0.02	0.13 ± 0.02	0.12 ± 0.03	0.08 ± 0.03
	HAL	0.25 ± 0.03	0.19 ± 0.02	0.17 ± 0.02	0.10 ± 0.02	0.10 ± 0.03	0.13 ± 0.04
− 4.00 to − 4.875	OK	0.23 ± 0.03	0.20 ± 0.03	0.16 ± 0.02	0.11 ± 0.03	0.08 ± 0.03	0.09 ± 0.03
	HAL	0.28 ± 0.04	0.21 ± 0.03	0.21 ± 0.03	0.12 ± 0.03	0.09 ± 0.04	0.12 ± 0.04
− 5.00 to − 5.875	OK	0.23 ± 0.05	0.14 ± 0.04	0.17 ± 0.03	0.05 ± 0.03	0.09 ± 0.03	0.11 ± 0.06
	HAL	0.32 ± 0.06	0.25 ± 0.07	0.26 ± 0.04	0.22 ± 0.04	0.12 ± 0.03	0.08 ± 0.05
− 6.00 to − 9.00	OK	0.16 ± 0.14	0.14 ± 0.06	0.22 ± 0.06	0.00 ± 0.06	0.04 ± 0.06	0.10 ± 0.08
	HAL	0.26 ± 0.06	0.22 ± 0.05	0.24 ± 0.05	0.25 ± 0.04	0.16 ± 0.03	–

*AL* = axial length; *D* = diopter; *HAL* = highly aspherical lenslets; *OK* = orthokeratology; *SER* = spherical equivalent refraction

**Fig. 4:**
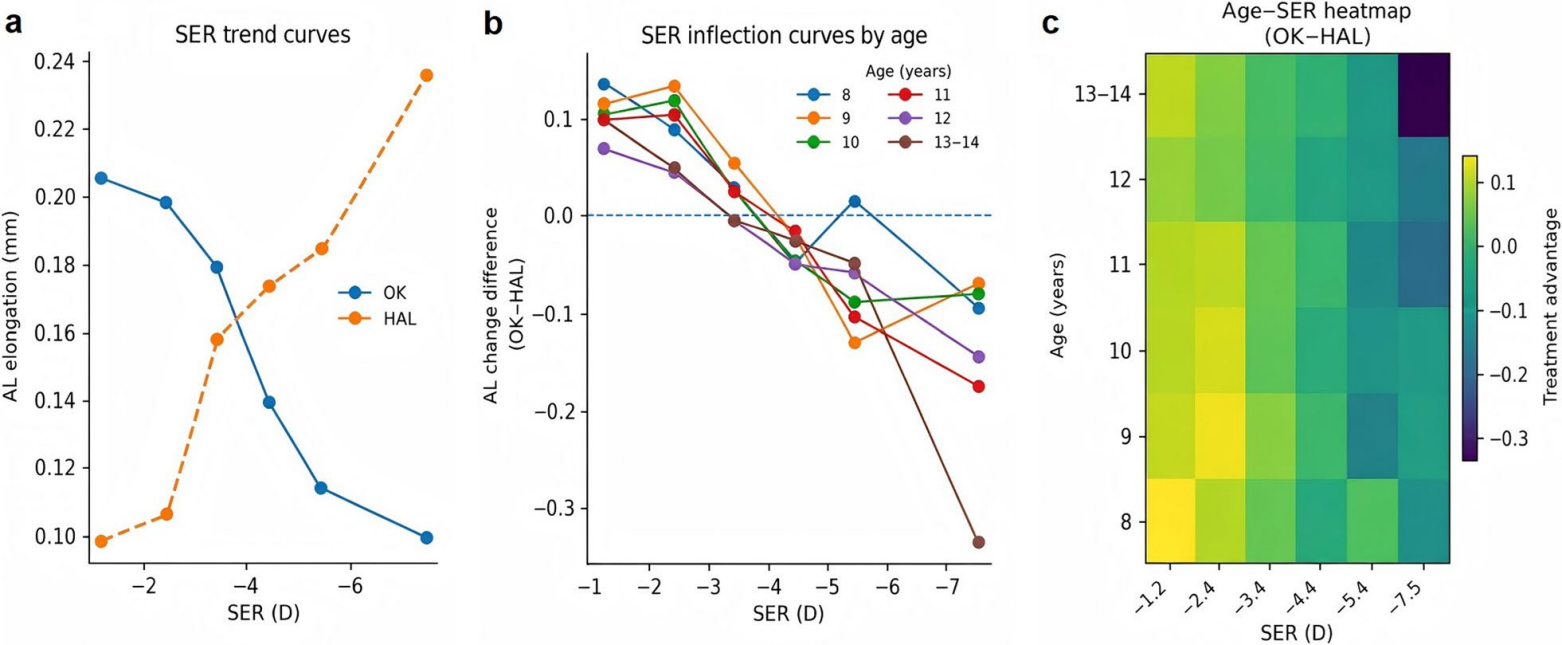
Age- and SER-specific patterns of AL elongation in OK and HAL. **a** Mean AL elongation trends across the SER spectrum in the OK and HAL groups. **b** Age-specific inflection curves showing the difference in AL elongation between treatments (OK–HAL) across SER levels. **c** Heatmap illustrating treatment advantage across age and SER strata, where positive values indicate smaller AL elongation in HAL and negative values indicate smaller AL elongation in OK. AL, axial length; HAL, highly aspherical lenslets; OK, orthokeratology; SER, spherical equivalent refraction

As shown in Fig. 4, the relationship between SER and AL elongation differed between the OK and HAL groups, and this pattern varied across age groups. Figure 4 illustrates age- and SER-specific patterns of AL elongation in the OK and HAL groups. Overall, AL elongation under OK decreased with increasing myopia, whereas AL elongation under HAL showed the opposite trend. Age-specific inflection curves showed that the difference between treatments (OK–HAL) was generally positive in eyes with lower myopia, indicating smaller AL elongation in the HAL group. As the SER became more myopic, the difference decreased and became negative in some age groups, suggesting a potential advantage of OK in highly myopic eyes. The heat map further demonstrated that HAL tended to show a greater benefit in children with lower myopia, whereas the difference between treatments diminished or reversed with increasing myopia and age.

Correlation analysis and multivariate linear regression models were further constructed in the matched cohort, with AL elongation as the dependent variable and age, sex, baseline SER, and baseline AL as independent variables.

**Fig. 5:**
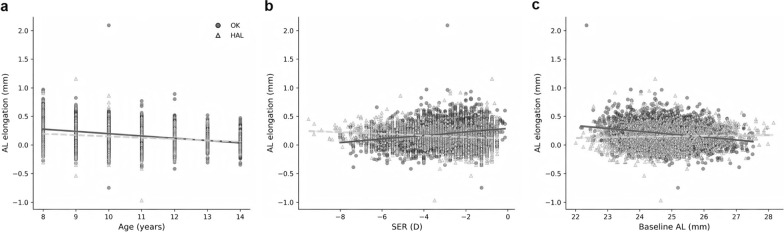
Associations between baseline factors and AL elongation. Relationship between baseline age and AL elongation (**a**), baseline SER and AL elongation (**b**), baseline AL and AL elongation (**c**). Scatter plots and fitted regression lines are shown separately for the OK and HAL groups. These plots illustrate the associations between baseline factors and subsequent AL elongation. AL, axial length; HAL, highly aspherical lenslets; OK, orthokeratology; SER, spherical equivalent refraction

In the OK group, AL elongation was significantly correlated with baseline age (r = − 0.373, *P* < 0.001), gender (r = 0.036, *P* = 0.044), baseline SER (r = 0.226, *P* < 0.001), and baseline AL (r = − 0.235, *P* < 0.001). In the HAL group, AL elongation also significantly correlated with baseline age (r = − 0.217, *P* < 0.001), sex (r = 0.037, *P* = 0.039), baseline SER (r = − 0.141, *P* < 0.001), and baseline AL (r = 0.048, *P* = 0.008). These associations are illustrated in Fig. 5.

Multivariate linear regression analysis demonstrated that the overall models were statistically significant in both groups (*P* < 0.001). In the OK group (R^2^ = 0.170), baseline age (β = − 0.335, *P* < 0.001), baseline SER (β = 0.142, *P* < 0.001), and baseline AL (β = − 0.052, *P* = 0.018) were independently associated with AL elongation, whereas sex was not significant (*P* = 0.132). Similarly, in the HAL group (R^2^ = 0.098), baseline age (β = − 0.298, *P* < 0.001), baseline SER (β = − 0.206, *P* < 0.001), and baseline AL (β = 0.056, *P* = 0.030) remained independent predictors of AL elongation, whereas gender did not reach statistical significance (*P* = 0.055) (Table 4).

**Table 4:** Correlation analysis and multivariable linear regression models for baseline factors associated with AL elongation (including baseline age, sex, baseline SER, and baseline AL)

Baseline parameters	Correlation analysis				Multivariable linear regression model (overall model: all *P* < 0.001)							
	OK		HAL		OK (R^2^ = 0.170)				HAL (R^2^ = 0.098)			
	r	*P*	r	*P*	B	95% CI	β	*P*	B	95% CI	β	*P*
Age (years)	− 0.373	< 0.001	− 0.217	< 0.001	− 0.036	− 0.040 to − 0.032	− 0.335	< 0.001	− 0.032	− 0.036 to − 0.028	− 0.298	< 0.001
Sex	0.036	0.044	0.037	0.039	0.010	− 0.003 to 0.023	0.027	0.132	0.013	− 0.000 to 0.026	0.037	0.055
SER (D)	0.226	< 0.001	− 0.141	< 0.001	0.020	0.014–0.025	0.142	< 0.001	− 0.022	− 0.027 to − 0.018	− 0.206	< 0.001
AL (mm)	− 0.235	< 0.001	0.048	0.008	− 0.011	− 0.021 to − 0.002	− 0.052	0.018	0.010	0.001–0.019	0.056	0.030

*AL* = axial length; *D* = diopter; *HAL* = highly aspherical lenslets; *OK* = orthokeratology; *SER* = spherical equivalent refraction

A comparison with previously published studies is summarized in Table 5. Across prior randomized controlled trials, both OK and HAL were associated with reduced AL elongation compared to single-vision correction, with reported effect sizes ranging from approximately 0.13–0.27 mm/year. In the present study, AL elongation was lower in the HAL group than in the OK group (0.15 mm/year vs. 0.20 mm/year), corresponding to a between-group difference of 0.049 mm/year.

**Table 5:** Comparison of the present study with previous studies on myopia control interventions

Study	Design	Age (years)	SER (D)	Intervention	Follow-up	AL elongation (control vs. intervention, mm/year)	Key findings
Cho et al. [5]	RCT	7–12	− 1.00 to − 4.00	OK vs. SV	2 years	0.29 vs. 0.54	OK significantly slowed AL elongation compared to SV
Cho et al. [4]	RCT	6–10	− 0.50 to − 4.00	OK vs. SV	2 years	0.36 vs. 0.63	Sustained AL control effect of OK
Lam et al. [19]	RCT	8–13	− 1.00 to − 5.00	DIMS vs. SV	2 years	0.17 vs. 0.55	DIMS significantly reduced AL elongation
Bao et al. [15]	RCT	8–13	− 0.75 to − 5.00	HAL vs. SV	1 year	0.13 vs. 0.36	HAL effective in controlling myopia progression
Present study	Retrospective (PSM)	8–14	− 0.50 to − 9.00	OK vs. HAL	1 year	0.20 vs. 0.15	HAL associated with less AL elongation than OK, with SER-dependent effects

*AL* = axial length; *DIMS* = defocus incorporated multiple segments; *HAL* = highly aspherical lenslets; *OK* = orthokeratology; *PSM* = propensity score matching; *RCT* = randomized controlled trial; *SER* = spherical equivalent refraction; *SV* = single-vision spectacles

## Discussion

The high prevalence of myopia and the increased risk of retinal diseases associated with high myopia have made it a global public health concern [1–3]. Various optical interventions are available [8]. This retrospective study evaluated AL elongation in children with myopia treated with OK and HAL in a real-world clinical setting. AL elongation differed between treatment modalities, and these differences were not uniform, but varied according to baseline age and refractive error. Specifically, HAL was associated with smaller AL elongation in younger children and in those with mild myopia, whereas OK was associated with smaller AL elongation in children with higher degrees of myopia. No significant differences were observed between older children and those with moderate myopia. Thus, the relative differences between treatments may depend on baseline clinical characteristics, particularly refractive status and age.

Previous studies have consistently demonstrated that both OK and myopia control spectacle designs are associated with reduced AL elongation compared with single-vision correction [4, 8–16, 19]. Reported annual AL elongation in children wearing OK lenses typically ranges from approximately 0.15–0.20 mm [4, 5, 6, 20], whereas values for HAL or similar defocus-incorporated spectacle designs generally range from 0.13 to 0.17 mm [15, 16, 19]. Thus, both treatment modalities were associated with the attenuation of AL elongation within a comparable range across different study designs and populations. The AL elongation observed in the present study (0.20 ± 0.18 mm in the OK group and 0.15 ± 0.17 mm in the HAL group) falls within these previously reported ranges, supporting the consistency of the current results with existing evidence. However, direct comparisons of OK and HAL remain limited, particularly across a wide spectrum of refractive errors. The present study extends the existing literature by demonstrating a refractive-error-dependent pattern of AL elongation. Specifically, the relative difference between treatments shifted across SER levels, with HAL showing smaller AL elongation in lower myopia and OK showing smaller AL elongation in higher myopia. This pattern may explain the inconsistencies across previous studies that did not stratify the results based on baseline refractive error [6, 7].

From a clinical perspective, the selection of an appropriate myopia management strategy remains challenging, particularly in children with varying degrees of refractive error. Previous studies have reported comparable AL elongation between OK and myopia-control spectacle designs in the general population [6]; however, these analyses often do not account for potential heterogeneity across refractive subgroups. In addition, variability in the study design, spectacle type, and inclusion criteria may have contributed to differences in the reported outcomes [6, 15, 19]. Furthermore, children with high myopia are frequently underrepresented in clinical studies due to fitting limitations or safety considerations, which may further limit their generalizability. This study addresses this gap by including a broader range of refractive errors and providing subgroup-specific comparisons.

One possible explanation for the observed refractive-error-dependent pattern may be related to the differences in peripheral optical defocus profiles between the treatment modalities. OK reshape the cornea and may induce peripheral myopic defocus, whereas HAL spectacles provide a relatively fixed level of peripheral defocus, typically between + 3.00 to + 5.00 D [15, 16]. Previous experimental and clinical studies have suggested that peripheral defocus plays an important role in axial growth [4, 21, 22]. It is plausible that in eyes with higher degrees of myopia, the greater refractive correction achieved with OK may result in a stronger or more extensive peripheral defocus signal, which could contribute to the observed differences in AL elongation. However, this interpretation remains speculative because peripheral optical characteristics and retinal image quality were not directly measured in this study. Future studies incorporating detailed optical modeling and biometric measurements are required to further elucidate these mechanisms.

The age-related differences observed in this study are consistent with the well-established age-dependent patterns of myopia progression. Younger children typically exhibit faster AL elongation that gradually slows with increasing age [1, 2, 3, 8, 9]. In the present study, the relative differences between the treatments were more pronounced in younger children and less evident in older children. Thus, age may influence the relative response to different optical interventions and underscores the importance of considering developmental stage when evaluating treatment outcomes. These findings support the rationale for early intervention in the management of myopia. From a clinical standpoint, treatment selection may benefit from an individualized approach based on the baseline refractive error and age. HAL may be associated with a smaller AL elongation in younger children and those with lower myopia, whereas OK may offer relative advantages in children with higher myopia. However, these findings should be interpreted cautiously because residual confounding from unmeasured variables (e.g., parental myopia, outdoor activity, near work, and treatment adherence) cannot be entirely excluded despite PSM and multivariable adjustment.

In untreated eyes, AL growth is age dependent, with faster elongation occurring in younger children and gradual deceleration with increasing age [1, 2, 3, 8, 9]. Children with higher degrees of myopia are often older on average and may therefore exhibit relatively slower axial growth. These interrelated factors may create overlapping and potentially confounding patterns when interpreting subgroup differences in age and refractive error. In the present study, these relationships were addressed through PSM and stratified analyses, which aimed to balance baseline age, refractive error, and AL between the groups. Despite these adjustments, a refractive error-dependent pattern in AL elongation remained evident, with differences between treatment modalities varying across the SER levels. Findings may be unlikely to be solely explained by the underlying age-related growth patterns. One possible explanation is that age may act not only as a confounder, but also as a biological modifier of treatment response. Younger eyes, which exhibit higher baseline growth rates, may be more responsive to certain optical signals, whereas structural and optical changes associated with greater refractive correction may alter the distribution and magnitude of peripheral defocus in eyes with higher myopia. Although this interpretation remains speculative, it highlights the importance of considering the combined effects of age and refractive status when evaluating AL elongation in children with myopia. Importantly, the direction of axial elongation across age subgroups remained consistent with the expected physiological patterns, supporting the biological plausibility of our findings.

The distribution of AL elongation values was examined and observed to be approximately normal across groups. The variability was relatively consistent across subgroups, as reflected by similar standard deviations. Therefore, the differences between the groups were primarily driven by shifts in the mean values rather than differences in dispersion (Fig. 2). A comparison with previous studies is summarized in Table 5, which highlights the differences in the study design, participant characteristics, and AL elongation outcomes. While randomized controlled trials provide high-quality evidence under controlled conditions, the present study contributes complementary real-world data by directly comparing OK and HAL in routine clinical practice, thereby enhancing the applicability of the findings.

This study has some limitations. First, the sample size in the high myopia subgroup (SER, − 5.00 to − 9.00 D) was relatively small (OK: n = 228; HAL: n = 345), which may have reduced the statistical power and increased uncertainty in the subgroup estimates. Second, as a retrospective study, treatment allocation was not randomized, and residual confounding from unmeasured variables (e.g., parental myopia, outdoor exposure, near-work activities, and treatment adherence) could not be entirely excluded despite the use of PSM and multivariable adjustment. Third, AL elongation was assessed based on the total AL without distinguishing specific ocular components, such as vitreous chamber depth. Fourth, the potential influence of the optimized OK designs was not evaluated [23, 24]. In addition, children with high myopia may have been more likely to receive alternative or combination therapies, which may have affected subgroup representativeness. Fifth, the follow-up period was limited to 12 months; long-term studies are required to determine the sustainability of these findings. Finally, in participants with high myopia, the residual refractive error after OK lens removal may fluctuate throughout the day, making precise spectacle correction difficult. Therefore, supplementary correction may only approximate refractive status and introduce variability in AL elongation outcomes, representing a potential source of residual confounding factors. This limitation is inherent in real-world practice and may introduce variability in the AL elongation outcomes. Nevertheless, AL elongation in the high myopia OK group remained relatively low, which is consistent with previous findings [17], suggesting that the combined approach may still provide effective control. This finding is also consistent with broader clinical evidence supporting orthokeratology for myopia control [25, 26]. Furthermore, a recent two-year study directly compared the myopia-control efficacy of HAL and OK lenses [27].

## Conclusion

AL elongation differed between the OK and HAL groups, and these differences varied according to age and baseline refractive error. HAL was associated with a smaller AL elongation in younger children and those with lower myopia, whereas OK was associated with a smaller AL elongation in children with higher myopia. The relative differences between treatment modalities were not uniform and may depend on the baseline clinical characteristics, which may have implications for individualized myopia management strategies.

## Data Availability

Te data used to support the fndings of this study are available from the corresponding author (Xinjie Mao) upon request.

## Abbreviations

SER: Spherical equivalent refraction
AL: Axial length
OK: Orthokeratology
HAL: Highly aspherical lenslets
PSM: Propensity score matching
